# Quorum Sensing: An Under-Explored Phenomenon in the Phylum *Actinobacteria*

**DOI:** 10.3389/fmicb.2016.00131

**Published:** 2016-02-10

**Authors:** Ashish V. Polkade, Shailesh S. Mantri, Umera J. Patwekar, Kamlesh Jangid

**Affiliations:** Microbial Culture Collection, National Centre for Cell Science, Savitribai Phule Pune University CampusPune, India

**Keywords:** Actinobacteria, *Streptomyces*, *Mycobacterium*, quorum sensing, GBL, MMFs, c-di-GMP, quorum quenching

## Abstract

Quorum sensing is known to play a major role in the regulation of secondary metabolite production, especially, antibiotics, and morphogenesis in the phylum *Actinobacteria*. Although it is one of the largest bacterial phylum, only 25 of the 342 genera have been reported to use quorum sensing. Of these, only nine have accompanying experimental evidence; the rest are only known through bioinformatic analysis of gene/genome sequences. It is evident that this important communication mechanism is not extensively explored in Actinobacteria. In this review, we summarize the different quorum sensing systems while identifying the limitations of the existing screening strategies and addressing the improvements that have taken place in this field in recent years. The *γ*-butyrolactone system turned out to be almost exclusively limited to this phylum. In addition, methylenomycin furans, AI-2 and other putative AHL-like signaling molecules are also reported in Actinobacteria. The lack of existing screening systems in detecting minute quantities and of a wider range of signaling molecules was a major reason behind the limited information available on quorum sensing in this phylum. However, recent improvements in screening strategies hold a promising future and are likely to increase the discovery of new signaling molecules. Further, the quorum quenching ability in many Actinobacteria has a great potential in controlling the spread of plant and animal pathogens. A systematic and coordinated effort is required to screen and exploit the enormous potential that quorum sensing in the phylum *Actinobacteria* has to offer for human benefit.

## Introduction

**FIGURE 1:**
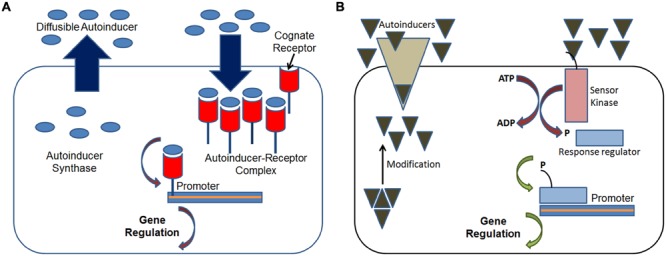
**Schematics of quorum sensing systems in bacteria. (A)** Gram-negative bacteria: at threshold concentrations the diffusible autoinducer signaling molecule, typically a homoserine lactone, binds to its cognate receptor inside the cell forming the autoinducer-receptor complex which then regulates the expression of target genes through its binding to the target promoter; and **(B)** Gram-positive bacteria: at threshold concentrations the autoinducer peptide molecule which is actively transported activates the sensor kinase protein inside the cell which phosphorylates the response regulator protein, which then regulates the expression of target genes through its binding to the target promoter. Adapted from Koh et al. (2013).

Cell-to-cell communication in bacteria via quorum sensing is a density-dependent regulation of gene expression. The system relies on two major components, a signaling molecule and a transcriptional activator protein. In many Gram-negative bacteria, a member of the N-acylhomoserine lactone (AHL) family acts as a diffusible signal molecule, the synthesis of which is controlled by the members of the LuxI family of synthases (**Figure 1**). Above a threshold concentration, this signal molecule activates target genes in conjunction with a member of the LuxR family of transcriptional activators (Fuqua et al., 1996). The AHL-based quorum sensing system plays major role in regulating multiple functions such as bioluminescence (Nealson and Hastings, 1979), synthesis of antibiotics (Bainton et al., 1992), the production of virulence factors (Barber et al., 1997), exopolysaccharide biosynthesis (Beck von Bodman and Farrand, 1995), bacterial swarming (Eberl et al., 1996), and plasmid conjugal transfer (Fuqua and Winans, 1994). In contrast, most Gram-positive bacteria use processed oligo-peptides for signaling and communication (Kleerebezem et al., 1997; Sturme et al., 2002). These signals, referred to as autoinducing polypeptides (AIPs) are produced in the cytoplasm as precursor peptides and are subsequently cleaved, modified, and exported. The AIP-based quorum-sensing systems are known to regulate the expression of many factors such as genetic competence (Solomon et al., 1995), sporulation (Magnuson et al., 1994), and virulence factor expression (Qin et al., 2000). While it may seem that the differentiation in the type of signaling compound is a consequence of the structural differences in the cell wall between the two bacterial types; however, this is not the case. For instance, certain Actinobacteria (Gram-positive) are known to use γ-butyrolactones for signaling, whereas most Gram-negative bacteria are known to possess signaling peptides as part of their genome (Lyon and Novick, 2004). Regardless of the cell type, quorum sensing is a near universal mode of cell-to-cell communication amongst pathogenic bacteria. Hence, it is now considered an important target for controlling their spread, especially antibiotic resistant bacteria.

Despite the diversity and importance of the phenotypes that are regulated by the quorum sensing network, the information on their environmental distribution is very limited. Further, those that are available, only focus on the AHL-mediated gene expression systems. A survey by Manefield and Turner (2002) showed that merely 2.2% (21 bacterial genera) of the total number of bacterial genera listed in the *Bergey’s Manual of Systematic Bacteriology* (Garrity et al., 2001) are known to harbor the AHL producing species, and all of which belong to the alpha, beta and gamma proteobacteria only. At the species level, the percentage of AHL producers drops to a fraction of a percent. Although the estimate is more than a decade old, it still reflects on the state of the information available on quorum sensing in bacteria. Motivated by this lack of information, our screening for *luxRI* homologs and AHL production in the genus *Aeromonas* not only revealed that the homologs are universally present in this genus, but also reported that a wide diversity of AHLs are secreted by the species in the genus (Jangid et al., 2007, 2012). This study only points to the fact that quorum sensing is indeed a widespread phenomenon among bacteria, however, a systematic evidence is lacking. Thus, there is a need to survey the existence and study the taxonomic distribution of the quorum sensing systems amongst bacterial taxa.

The phylum *Actinobacteria* is one of the largest phyla within domain *Bacteria* and consists of six classes, 23 orders including one *Incerta sedis* and 53 families (Ludwig et al., 2012). As of December 2015, there were 342 genera in this phylum with standing in nomenclature as determined from the LPSN database (Parte, 2015). Actinobacteria are typically Gram-positive but at times stain-variable and have a rigid cell wall that contains muramic acid with some containing wall teichoic acids. The phylum comprises of a plethora of phenotypically diverse organisms, with widespread distribution in nature and exhibiting varied oxygen, nutritional, temperature, and pH requirements for growth, making it an important phylum.

Their diverse physiological potential makes Actinobacteria a dominant role player in the biotechnology industry. Their applications are widespread and vary from agroindustry, pharmaceuticals, bioremediation among numerous others. They play a key role in natural geochemical cycles, especially through their ability to decompose organic matter. Actinobacteria are also abundant in the rhizosphere and produce a wide range of biologically active metabolites, thereby influencing plant development (Selvakumar et al., 2014). Many Actinobacteria are also known pathogens of plants and animals. However, amongst the most important potential of Actinobacteria, it is the production of a significant number of secondary metabolites like antibiotics and other compounds of biotechnological interest that has been exploited most. For instance, among the polyene macrolides, a class of polyketides which are antifungal compounds, are synthesized by more than 100 different species of actinomycetes (Recio et al., 2004). In addition, members of the genus *Streptomyces* are known to produce more than 70% of commercially available antibiotics (Weber et al., 2003). The expression of virulence determinants, production of secondary metabolites, and morphogenesis is associated with high cell densities and typically controlled by diffusible low molecular weight chemical substances, similar to the Gram-negative autoinducer, suggesting a role of quorum sensing in regulating these mechanisms (Takano, 2006; Santos et al., 2012). Further exploration of novel phenotypes under quorum sensing regulations is likely to contribute to the advancement in medical, biotechnological and ecological fields. Hence, there is a need of studying quorum sensing in *Actinobacteria.*

Most of what is known about quorum sensing in Actinobacteria, comes from the study of antibiotic production in this taxa. While it is indeed the most important phenomenon, the aim of this review is not to present an overview on the quorum sensing regulation of antibiotic production. The reader is therefore directed to read Takano (2006), Liu et al. (2013) and references within. Further, for clarity Actinobacteria means all species within the phylum *Actinobacteria*, unless otherwise stated as class *Actinobacteria.*

In this review, we present an overview of quorum sensing systems described so far for the phylum *Actinobacteria*, indicating the limitations of existing screening strategies and addressing improvements in newer technologies for the discovery of quorum sensing in more taxa. In addition, we summarize the current status of known quorum quenching activity in this phylum.

## Quorum Sensing in the Phylum *Actinobacteria*

**FIGURE 2:**
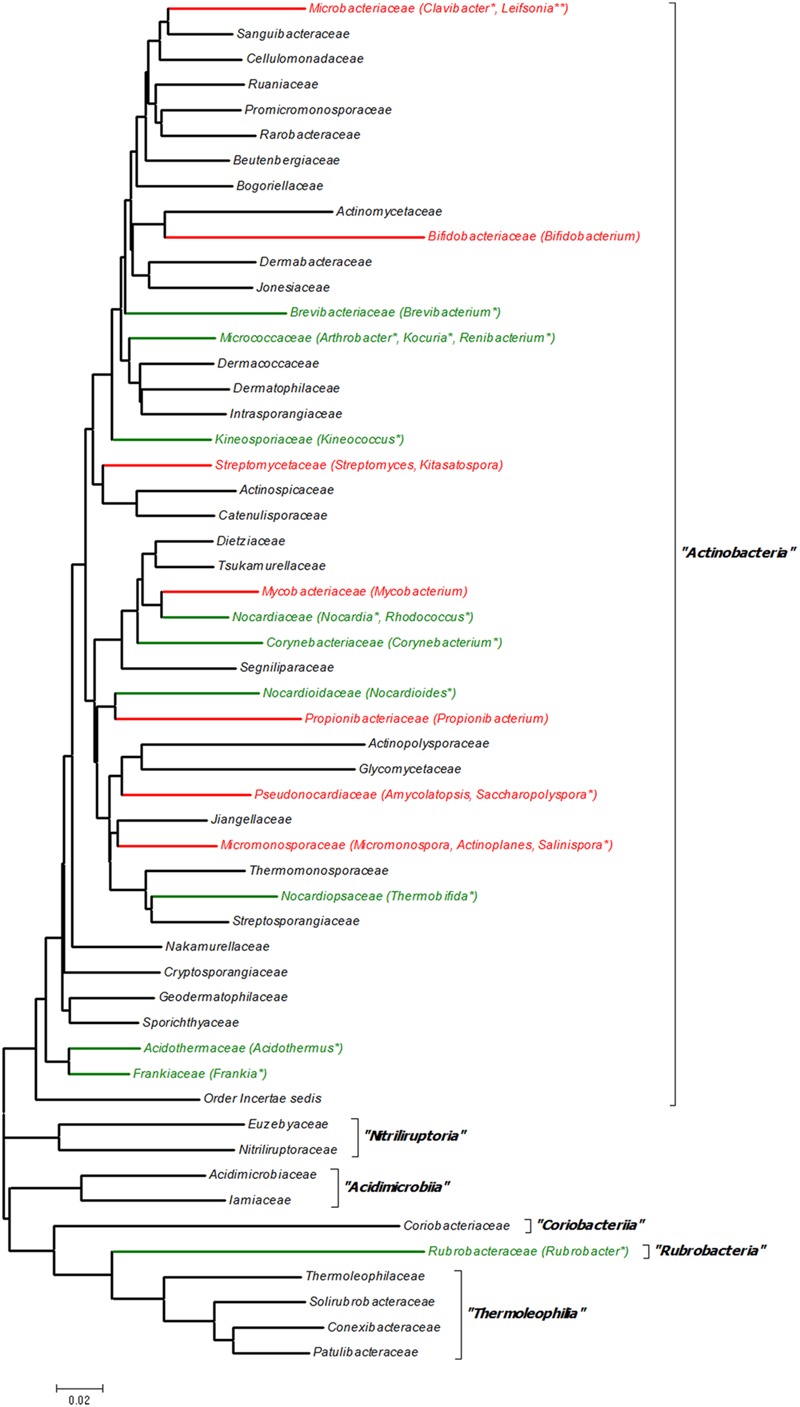
**16S rRNA gene sequence based family tree of the phylum *Actinobacteria* depicting the genera with known quorum sensing systems.** The evolutionary history was inferred using the Neighbor–Joining method (Saitou and Nei, 1987) using the bootstrap test of phylogeny (1000 replicates) (Felsenstein, 1985). The evolutionary distances were computed using the Kimura 2-parameter method (Kimura, 1980) Evolutionary analyses were conducted in MEGA6 (Tamura et al., 2013). A total of 54 sequences were used for constructing the tree and belonged to the type species of the type genera of each family as described in Ludwig et al. (2012) and are submitted as Supplementary Data in fasta format. Black color branch denotes family with no evidence of quorum sensing; red color denotes family with experimental evidence of quorum sensing; green color denotes family for which only gene homologs are known; ‘^∗^’ indicates genus for which only gene homologs are known; and ‘^∗∗^’ denotes genus for which putative AHL-like signal molecules are involved in quorum sensing.

Although Actinobacteria is one of the largest groups of organisms in the bacterial domain, very few reports were available for known quorum sensing regulation in the phylum. An analysis of literature for quorum sensing in Actinobacteria revealed that only 25 actinobacterial genera have some sort of quorum sensing regulation (**Figure 2**). This number represents a mere 7.3% of the 342 genera reported in the latest update of LPSN (Parte, 2015). Of these, only nine genera (2.6%) have known quorum sensing regulation, whereas remaining 16 genera (4.7%) are known to only harbor the homologs of LuxR based on the analysis of available gene/genome sequences. It is noteworthy that 24 of the 25 genera belonged to the single class *Actinobacteria* whereas only a single genus *Rubrobacter* belonged to the class *Rubrobacteria.* No reports were available for the other four actinobacterial classes: *Acidimicrobiia, Coriobacteriia, Nutriliruploria*, and *Thermoleophilia*. This short list in fact suggests that an enormous scope exists for screening more taxa for further exploration of quorum sensing in Actinobacteria.

One quorum sensing system that seems to be limited to Actinobacteria is the *γ*-butyrolactone (GBL) system. The GBL system is quite similar to the AHL-based system in Gram negative bacteria due to the structural similarity between GBL and AHL, as well as that it is a one-component system where the communication molecule sensing protein is also the response regulator (Takano, 2006). Most reports on the GBL-system come from the genus *Streptomyces* which produces numerous important secondary metabolites and undergoes a sophisticated morphological differentiation program (Horinouchi and Beppu, 1993; Takano et al., 2000) (**Table 1**). In most cases, these processes are under the direct control of GBL autoregulator in tandem with specific cognate GBL receptors (Healy et al., 2009). The membrane-diffusible GBL autoregulator controls the expression of structural genes encoding secondary metabolite pathway enzymes. The GBL receptors are transcriptional regulators belonging to the TetR superfamily of transcription factors (Nishida et al., 2007). Given the large number of species in the genus *Streptomyces* and the very few GBL regulatory systems known, lot more work on the signaling cascade and receptor proteins is required.

**Table 1:** Status of quorum sensing systems in *Actinobacteria*.

S. No.	Genus	Signal type	Proteins/Homologs Involved/Domain Architecture	Phenotypes regulated	Reference
**Actinobacterial genera with experimental evidence of quorum sensing**					

1	*Actinoplanes*	VB-type		Medically important secondary metabolite	Choi et al., 2003
2	*Amycolatopsis*	IM2-type		Medically important secondary metabolite	Choi et al., 2003
3	*Bifidobacterium*	Autoinducer AI-2	LuxS, LuxR_C_Like, REC	Biofilm formation	Santos et al., 2012; Sun et al., 2014
4	*Kitasatospora*	GBL	KsbA	Bafilomycin production	Choi et al., 2004
5	*Leifsonia*	Putative AHL signal	LuxR_C_Like, REC		Santos et al., 2012; This study
6	*Micromonospora*	IM2-type with long C2 chain		Medically important secondary metabolite	Choi et al., 2003
7	*Mycobacterium^∗^*	cAMP and cGMP, ppGpp, c-di-GMP and c-di-AMP	AAA, CHD, HDc, LuxR_C_Like, MAP0928, REC, WhiB3	Biofilm formation and pathogenicity	Banaiee et al., 2006; Takano, 2006; Chen and Xie, 2011; Santos et al., 2012; Sharma et al., 2014
8	*Propionibacterium*	Autoinducer AI-2	LuxR_C_Like, REC	Biofilm formation and upregulation of virulence factors	Coenye et al., 2007; Santos et al., 2012; Lwin et al., 2014
9	*Streptomyces^∗^*	GBLs, MMFs, Factor-A, Factor-I, IM-2, VB, PI factor	AAA, AlpZ, AplW, ArpA, Aur1R, AvrA, BarA, BarB, Brp, CprA, FarA, JadR2, LuxR_C_Like, MmfR, NcsR2, Orf74, Orf79, Orf82, REC, SabR, SAV2268, SAV2270, SAV3702, ScbA, ScaR, ScbR, SCO6286, SCO6323, Sng, SpbR, TarA, TPR, TylP, TylQ	Production of antibiotics (Act, Clavulanic acid, Cephamycin, D-cycloserine, Kas, Methylenomycin, Natamycin, Nikkomycin, Nucleoside, Pristinamycin, Red, Streptomycin, Tylosin, Virginiamycin), morphogenesis and sporulation	Recio et al., 2004; Takano, 2006; Gottelt et al., 2012; Santos et al., 2012 (and references within); Willey and Gaskell, 2011

**Actinobacterial genera with only bioinformatic evidence of quorum sensing**					

10	*Acidothermus*		LuxR_C_Like, REC		Santos et al., 2012
11	*Arthrobacter^∗^*		AAA, LuxR_C_Like, REC		Santos et al., 2012
12	*Brevibacterium*		Transcriptional regulator (GenBank: ZP_00378009)		Takano, 2006
13	*Clavibacter*		LuxR_C_Like, REC		Santos et al., 2012
14	*Corynebacterium*		LuxR_C_Like, REC		Santos et al., 2012
15	*Frankia*		AAA, LuxR_C_Like, REC		Santos et al., 2012
16	*Kineococcus*		AAA, LuxR_C_Like, REC		Santos et al., 2012
17	*Kocuria*		LuxR_C_Like, REC		Santos et al., 2012
18	*Nocardia*		FHA, LuxR_C_Like, REC, Transcriptional regulator (GenBank: BAD59728, BAD55455)		Takano, 2006; Santos et al., 2012
19	*Nocardioides^∗^*		HDc, LuxR_C_Like, REC		Santos et al., 2012
20	*Renibacterium*		LuxR_C_Like, REC		Santos et al., 2012
21	*Rhodococcus^∗^*		AfsA, ArpA, CSP_CDS, FHA, HDc, LuxR_C_Like, PBD2.026, PKC, REC, TPR, Similar to VB-R (Genbank: AAR90230), Transcriptional regulator (GenBank: AAR90151)	Plant pathogenesis, Biocontrol agent	Takano, 2006; Wuster and Babu, 2008; Santos et al., 2012; Latour et al., 2013
22	*Rubrobacter*		LuxR_C_Like, PAS, REC		Santos et al., 2012
23	*Saccharopolyspora*		LuxR_C_Like, REC, SeaR, TPR		Takano, 2006; Santos et al., 2012
24	*Salinispora*		LuxR_C_Like, REC		Takano, 2006; Santos et al., 2012
25	*Thermobifida*		LuxR_C_Like, REC, TPR		Santos et al., 2012

*Information used in the table was derived from the references cited here and some taxa may have been missed. ‘^∗^’ denotes genera with known quorum quenching ability that also includes Microbacterium which is not shown in here as no known quorum sensing evidence exists for it.*

With the exception of the well characterized GBL-based system of *Streptomyces* sp. (Takano, 2006), communication in this phylum has been scarcely explored. Based mostly on indirect evidence, Santos et al. (2012) made a significant contribution toward increasing the number of genera known to harbor LuxR homologs. A diversified and stereoscopic organization of LuxR proteins among members of this phylum was reported through an extensive *in silico* analysis of the phylogenomic distribution and functional diversity of the LuxR proteins. The authors identified a total of 991 protein sequences from 53 species that contained at least one LuxR domain. The distribution of these sequences was not even among species and ranged from organisms with a single sequence (e.g., *Mycobacterium leprae*) to others with over 50 (e.g., *Streptomyces* sp.). Using a domain-based strategy, the LuxR family of proteins in Actinobacteria was shown to include two major subfamilies: one that resembled the classical LuxR transcriptional regulators and another in which the LuxR domain is associated with N-terminal REC domain (receiver). In a third and smaller group of sequences, LuxR domain appears associated with a series of signal transduction-related domains other than REC, forming multidomain proteins (Santos et al., 2012). From the evolutionary perspective, it was shown that the ancestor gene sequence codified for a protein with a single LuxR domain. The original LuxR-encoding genes then suffered a series of duplications presumably followed by functional specification, but they also acquired different domains, originating new subfamilies with implications in a wide range of functionalities. The phylogenetic results described suggested a conspicuous promiscuity of the LuxR domain among Actinobacteria. For details on the distribution of the LuxR proteins within the phylum, the reader is suggested to refer to the original study (Santos et al., 2012).

### Selective Actinobacteria with Known Quorum Sensing Systems

#### Streptomyces

The genus *Streptomyces* with 778 species (Parte, 2015) is the largest genus of *Actinobacteria* and is a natural inhabitant of soils and decaying vegetation. *Streptomyces* are characterized by its complex morphological differentiation and their ability to produce a variety of secondary metabolites, contributing to two-thirds of naturally occurring antibiotics. The synchronized behavior of these species in producing antibiotics and modulation of gene expression is governed by quorum sensing through a spectrum of small chemical signaling molecules, called GBLs (Bhukya et al., 2014). GBLs diffuse freely through the cell membrane and regulate these pathways when the intra and extracellular concentrations of GBLs reaches a threshold. In this sense, they behave very similar to the AHL-based quorum sensing in Gram-negative bacteria.

**FIGURE 3:**
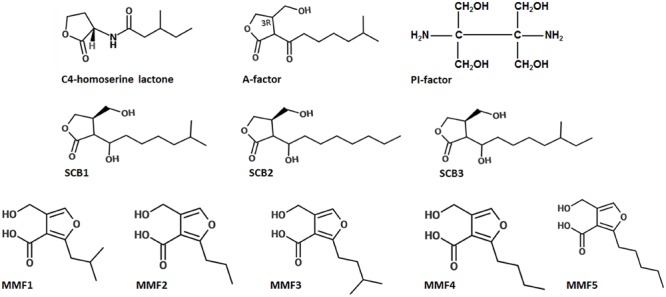
**Structures of representative signaling molecules in *Actinobacteria*.** The A-factor of *Streptomyces griseus*, the GBLs of *S. coelicolor* (SCB1, SCB2, and SCB3), MMFs of *S. coelicolor* which are structurally distinct sharing a common 2-alkyl-4-hydroxymethylfuran-3-carboxylic acid core structure but differ in the identity of the C2 alkyl group. The C4-homoserine lactone of *Pseudomonas aeruginosa* is shown for comparison. Adapted from Willey and Gaskell (2011).

Much of what is known in actinobacterial quorum sensing could be attributed to the information gained from GBL-based quorum sensing in *Streptomyces.* In fact, the first signaling molecules, the GBLs, were already known from *Streptomyces* in the 1960s (Khokhlov et al., 1967) much before the term ‘quorum sensing’ was coined by Fuqua et al. (1994). Today, at least 60% of *Streptomyces* species appear to produce GBLs regulating multiple phenotypes even in nano Molar concentrations (Takano et al., 2000). Most GBLs are structurally similar but chemically distinct. They are extractable in organic solvents and are heat, protease, and acid resistant. Although they share structural similarity with AHLs (except for the carbon side-chain, **Figure 3**), the GBL receptors do not bind to AHL or vice-versa. At the genomic level, a lineage-specific LuxR protein homolog with a very limited diversity of associated domains is known to exist in *Streptomyces* (Santos et al., 2012).

**FIGURE 4:**
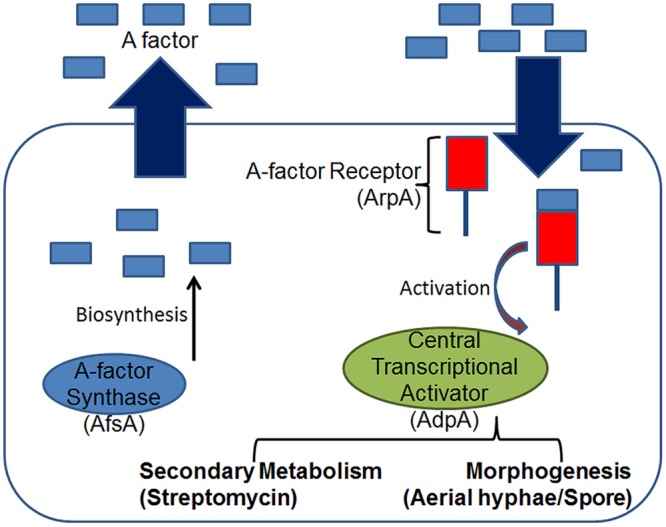
**The *S. griseus* A-factor regulon.** Similar to Gram-negative bacteria, at threshold concentrations the diffusible A-factor (a γ-butyrolactone) binds the intracellular receptor ArpA and activates expression of the transcriptional activator AdpA which in-turn regulates multiple phenotypes either indirectly via a multi-step cascade, such as the development of aerial hyphae and sporulation, or directly, such as the production of secondary metabolites like streptomycin.

Their molecular mechanism reveals a diverse and complex system (Choi et al., 2003). The most intensively studied GBL is A-factor or the autoregulatory-factor (2-isocapryloyl-3*R*-hydroxymethyl-g-butyrolactone), which is known to control the expression of more than a dozen genes, amongst which streptomycin production and sporulation in *Streptomyces griseus* are the most extensively studied (Takano et al., 2000) (**Figure 4**). It is known to exert its effects on both clonal hyphae in a single mycelium as well as genetically distinct *S. griseus* hyphae. A-factor likely diffuses between filaments and acts by biding with the A-factor receptor, ArpA which is a transcriptional repressor that targets *adpA*. Upon binding, the A-factor-ArpA complex activates *adpA* expression (Willey and Gaskell, 2011). A suit of genes are under the AdpA-dependent activation, such as *strR* whose expression regulates the streptomycin biosynthetic gene cluster, and genes that are involved in morphological differentiation. All the characteristics of A-factor tell us that A-factor is a microbial hormone comparable to eukaryotic hormones such as the sex pheromones controlling differentiation in fungi (Horinouchi and Beppu, 1993). However, it is neither the most abundant nor the most stable GBL. The *S. coelicolor* butanolide 1 (SCB1), reported previously to stimulate blue-pigmented polyketide actinorhodin (Act) and the red-pigmented tri-pyrolle undecylprodigiosin (Red) production in a growth phase-dependent manner, is known to be most abundant and more stable than A-factor (Takano, 2006). The genes involved in the synthesis of SCB1 (*scbA*) and binding (*scbR*) have been identified (Gottelt et al., 2012). ScbR regulates transcription of both scbA and itself by binding to the divergent promoter region controlling both genes, and the GBL SCB1 inhibits this binding. *S. coelicolor* is known to produce multiple GBLs with distinct biological activities. Similarly, *S. virginiae* produces at least five virginiae butanolides (VB-A, B, C, D, and E) that stimulate virginiamycin production, each with a different minimum effective concentration. In contrast, both *S. griseus* and *S. lavendulae* produce a single GBL, the A-factor and IM-2, respectively. While A-factor regulates streptomycin production in *S. griseus*, IM-2 regulates the production of nucleoside antibiotics showdomycin and minimycin in *S. lavendulae* (Gottelt et al., 2012). The presence of multiple GBLs in *Streptomyces* is an indication of the complex communication mechanisms that exist in this genus and have still not been explored in great details.

A new class of water soluble autoinducer different from the GBLs was reported by Recio et al. (2004). This factor, called the PI Factor was identified as 2,3-diamino-2,3-bis(hydroxymethyl)-1,4-butanediol (**Figure 3**). It was isolated from *S. natalensis* and regulates Pimaricin biosynthesis in the organism. By using complementation assay, pimaricin production was restored in the presence of the A-factor in a pimaricin-impaired mutant. Similar to other GBLs, the PI factor is also active at nano Molar concentrations. However, the restoration of pimaricin production in the presence of both A-factor and PI factor suggests that *S. natalensis* has an integration of multiple quorum signals from actinomycetes. Interestingly, the PI factor has not been reported in the microbial world and has an entirely novel chemical structure that is only distantly related to the homoserine lactone and furanosyl diester inducer families. These unique properties of PI factor only point to the fact that this taxa holds a great potential for further exploration of quorum sensing.

In addition to GBLs, methylenomycin furans (MMFs) have recently been shown to regulate antibiotic production in *S. coelicolor* via quorum sensing (Willey and Gaskell, 2011). Different *S. coelicolor* mutants that were deficient in methylenomycin production, would co-synthesize the antibiotic when grown in close proximity of each other, suggesting that a diffusible signal was involved in its biosynthesis. Between the two, the mutant strain that rescued the non-producer is called the ‘secretor’, whereas the one that regained the capacity to produce the antibiotic when grown near secretor is called the ‘convertor’. Studies have shown that the secretor strains possess the ability to synthesize small signaling molecules similar to GBLs, called MMFs, but themselves lack the methylenomycin biosynthetic genes, while the opposite is true of converters. While being very similar to GBLs in chemical properties, the MMFs are structurally distinct with a common 2-alkyl-4-hydroxymethylfuran-3-carboxylic acid core but a different C2 alkyl group (**Figure 3**). The discovery of MMFs only points to the fact that the exploration of quorum sensing in Actinobacteria is very limited and the possibility of discovering such novel homologs is not farfetched, it just needs a systematic approach (Willey and Gaskell, 2011).

#### Mycobacterium

Mycobacteria hold an extreme medical importance worldwide. *Mycobacterium tuberculosis* is a successful human pathogen, with ∼2 × 10^9^ individuals; nearly one-third of the world’s population infected globally (Banaiee et al., 2006). The distinguishing feature of mycobacteria is the presence of thicker cell wall which is rich in mycolic acids and a very slow growth rate. With the emergence of drug-resistance, treating mycobacterial infections is becoming increasingly difficult and hence, looking for newer drug targets, especially those involving quorum sensing is an essential component of mycobacterial research. However, the Gram positive mycobacteria remain a mystery with no clear evidence known about their quorum sensing mechanism (Sharma et al., 2014). Bioinformatics analysis has revealed the presence of LuxR homologs in *M. tuberculosis*, but the experimental supports are lacking (Chen and Xie, 2011; Santos et al., 2012). Some of these homologs are ubiquitous across the multiple mycobacterial species and are involved in mycobacterial biofilm formation or persistence, suggesting a possible existence of similar quorum sensing mechanisms. Given the fact that biofilm formation is mostly linked with quorum sensing regulation and with many non-tuberculous mycobacteria known to form biofilms, such as *M. smegmatis, M. marinum, M. fortuitum, M. chelonae, M. ulcerans, M. abscessus, M. avium*, and *M. bovis* (Sharma et al., 2014), the existence of quorum sensing in these organisms cannot be ruled out. However, this hypothesis needs experimental validation.

The evidence of quorum sensing in Mycobacteria is mostly indirect. The *M. tuberculosis whiB3* gene, a putative transcriptional regulator that was recently implicated in causing gross and microscopic lesions, is likely to be under quorum sensing regulation (Banaiee et al., 2006). Although no evidence was presented, the expression pattern of *whiB3* was shown to reflect changes in bacterial density thereby suggesting a role for quorum sensing in its regulation. A survey of 22 *M. tuberculosis* genes showed that *whiB3* was induced maximally during the early phase of infection in the mouse lung and cultured macrophages. The expression of *whiB3* inversely correlated with bacterial density in the mouse lung, BMMϕ medium, and broth culture (Banaiee et al., 2006). Since this pattern of expression is consistent with quorum sensing, further studies are warranted to study this system in *M. tuberculosis*.

Another indirect evidence of the involvement of quorum sensing regulation in mycobacteria is known through the studies on second messengers. Second messengers are those compounds that are involved in the signal transduction phosphorelay cascade enabling the ‘decoding’ of the ‘coded’ information received in the form of quorum sensing molecules (autoinducers) to sense and bring appropriate changes in their environment by expression of target genes (Bharati and Chatterji, 2013). Thus, inter- and intra-cellular signaling must be integrated. A variety of small molecules, such as, mono (cAMP and cGMP) and di-cyclic or modified nucleotide (ppGpp, c-di-GMP, and c-di-AMP), are important intracellular signaling molecules in mycobacteria and play a key role in relaying the signals received from the receptor (on the surface) to the target molecule in the cell (Sharma et al., 2014). These nucleotide-based second messengers regulate different processes in various bacterial systems. Of these, c-di-GMP is a ubiquitous bacterial second messenger and in effective concentrations it is known to facilitate phenotypes, such as virulence and biofilm formation. The involvement of these second messengers indirectly implies the existence of quorum sensing systems in both the pathogenic and non-pathogenic mycobacteria (Sharma et al., 2014).

#### Propionibacterium

*Propionibacterium acnes* is an anaerobic Gram-positive rod shaped bacterium which is a natural inhabitant of human skin. It plays an important role in the pathogenesis of acne vulgaris, a common disorder of the pilosebaceous follicles. However, as the infection progresses the organisms shows resistance to antibiotics. In fact, there has been a gradual decrease in the efficacy of topically applied erythromycin, most likely due to the development of resistance via biofilm formation. Indeed, genomic analysis of *P. acnes* shows that the organism has three separate gene clusters that code for enzymes involved in extracellular polysaccharide biosynthesis, suggesting that it is capable of forming the necessary extracellular biofilm matrix (Coenye et al., 2007). Further experimentation revealed that the organism is able to form biofilms as well as showed increased production of the autoinducer AI-2 by sessile cells of *P. acnes* and the upregulation of its virulent activity, such as hydrolysis of sebum triglycerides by its bacterial lipases, secreting free fatty acids (FFAs) such as oleic, palmitic, and lauric acids. The increased concentration of such irritant fatty acids is thought to contribute to the inflammation and thereby plays an important role in the pathogenesis of acne. While the discovery of AI-2 suggested the presence of quorum sensing in this organism, the mechanisms under its regulation are still not clear.

In an interesting hypothesis, Lwin et al. (2014) proposed that quorum sensing indirectly plays a role in the pathogenesis of acne. Based on the danger model of immunity by Matzinger (1994) which states that responses to antigens are not dependent solely upon the recognition of ‘non-self’ by the immune system, initiation of the optimal immune response requires a sense of tissue damage or evidence of a pathogenic micro-organism via so called ‘danger signals’. In case of acne, the FFAs act as danger-associated molecular patterns. In its controlled growth as a skin commensal, *P. acnes* sends no or only ‘safety’ signals, but sends ‘danger’ signals via quorum sensing in the form of excess FFA production during pathogenic state, thereby stimulating inflammation. As of today, there is no *in vivo* evidence of quorum sensing by *P. acnes* even though a known quorum sensing signal, AI-2, is produced by the organism. However, experimental validation of this hypothesis is likely to offer novel therapeutic targets as well as open new possibilities of quorum sensing in this organism.

#### Rhodococcus

Actinobacteria in the genus *Rhodococcus* are aerobic, Gram-positive to variable and non-motile. They represent a group with remarkable metabolic diversity making them an ideal candidate for use in the bioremediation of contaminated sites, and as biocatalysts during biotransformations. Hence, they are of interest to the chemical, environmental, energy, and pharmaceutical sectors (Jones and Goodfellow, 2012). With a high economic value, further research into the exploration of physiological potential of this actinobacterial group is of increasing importance.

The presence of quorum sensing in *Rhodococcus* is only known through bioinformatic evidence based on genomic sequences of a few strains. Although GBL was detected in *Rhodococcus rhodochrous* NCIMB 13064 culture medium, it was shown that GBL accumulated due to chemical oxidation of haloalkane in high cell density cultures (Curragh et al., 1994). *In silico* analysis of the *Rhodococcus erythropolis* PR4 genome revealed the presence of genes encoding a communication molecule synthase, AfsA, and a communication molecule response regulator, ArpA with 31 and 36% amino acid sequence identity, respectively, suggesting the possible presence of a functional GBL-based quorum sensing system in this strain (Latour et al., 2013). A similar analysis of the genome of *Rhodococcus* strain RHA1showed the presence of homologs for protein domains of both the GBL synthase and the receptor which suggests that GBL might play a role in this organism too (Wuster and Babu, 2008). The fact that both the synthase genes and the response regulator genes are in close proximity of each other as in the case of AHL-based quorum sensing systems, suggests that these homologs may act as a quorum sensing system. This suggests that such a system is present in *Rhodococcus*.

#### Bifidobacteria

With a substantial effort in categorizing the human microbiome, new information has revealed that members of the genus *Bifidobacteria* represent one of the dominant groups of normal human gastrointestinal microbiota. They are also among the first colonizers of the gastrointestinal tract after birth. At the genomic level, all publically available genome sequences of bifidobacteria harbor putative *luxS* genes, and their corresponding amino acid sequences are well conserved in the genus with >82% sequence similarity to the LuxS protein of *Vibrio harveyi* (Sun et al., 2014). Using this information, Sun et al. (2014) experimentally confirmed that *Bifidobacteria* sp. exhibit LuxS-dependent AI-2 activity and biofilm formation. In this context, AI-2- dependent biofilm formation, e.g., on food particles or host-derived mucus, could be an important mechanism for early colonization of the host by commensal strains or persistence for prolonged periods by probiotic strains (Sun et al., 2014). With major implications in human health due to their use as probiotics, the advantages of exploiting the biofilm formation capability in bifidobacteria are enormous.

#### Other Actinobacteria

The evidence of quorum sensing in other actinobacterial genera is very meager. At least three closely related non-*Streptomyces* genera are known to produce GBL autoregulators and their receptor proteins based on specific ligand-binding assay (Choi et al., 2003). Using the binding assay with tritium-labeled autoregulator analogs as ligands, the authors screened crude cell-free lysates of five different non-*Streptomyces* strains with intermittent samplings during cultivation up to 96 h. The authors concluded that the teicoplanin-producer *Actinoplanes teichomyceticus* IFO13999 produced a VB type autoregulator, whereas both the rifamycin- producer *Amycolatopsis mediterranei* IFO13415 and the gentamicin-producer *Micromonospora echinospora* IFO13150 produced IM-2-type autoregulators. However, the IM-2 autoregulator produced by *M. echinospora* was likely with a longer C2 side chain as the biosensor strain *S. lavendulae* FRI-5 only recognizes IM-2-type autoregulators having a C2 side-chain length of 4–5 carbons (Choi et al., 2003). Moreover, the production of the autoregulators roughly corresponded to the late exponential growth phase and reached a plateau between 48 and 60 h, at the early stationary phase. The inability to detect autoregulator(s) in the other two strains, *Actinoplanes* sp. ATCC 31044 and *Amycolatopsis orientalis* IFO12806, does not exclude the possibility that, under different conditions, these strains might produce autoregulator(s). Hence, screening for autoregulators in different conditions using multiple biosensors is the key to go ahead in future.

Among other Actinobacteria, evidence exists for *Frankia* and *Nocardia* from genome analysis that they possess homologs of AfsA and ArpA, respectively (Wuster and Babu, 2008). Similarly, a LuxR system including a putative two-component system response regulator of the LuxR family of protein together with 23 transcriptional regulators is reportedly present in the *Nocardia brasiliensis* HUJEG-1 as determined based on its complete genome sequence (Vera-Cabrera et al., 2013). Further, members of the genera *Acidothermus, Arthrobacter, Brevibacterium, Clavibacter, Corynebacterium, Kineococcus, Kocuria, Nocardoides, Renibacterium, Rubrobacter, Saccharopolyspora, Salinispora*, and *Thermobifida* are known to harbor homologs of the LuxR regulators (Takano, 2006; Santos et al., 2012). Although LuxR regulators may also be involved in intracellular signaling, the presence of LuxR proteins is intriguing since no AHLs are known to act on the actinobacterial quorum sensing systems, where signaling is generally assured by cyclic or modified peptides and GBLs. However, this does not exclude the possibility that AHL-mediated quorum sensing does not exist in Actinobacteria because none of the screening strategies reported till date have used the conventional AHL-responsive biosensors. While our preliminary screening for AHL-mediated quorum sensing in Actinobacteria using AHL-responsive biosensors yields support for this hypothesis (personal observation), further investigation would help in ascertaining whether certain actinobacterial strains release AHLs or if there are other as-yet unknown compounds to which these AHL-responsive biosensors respond. In either case, AHL-production by Actinobacteria or AHL-responsive biosensors responding to the non-AHL signals produced by Actinobacteria is interesting. In this context, Yang et al. (2009) noted that *N*-hexanoyl-DL-homoserine lactone (C_6_-HSL) interacts with the *S. coelicolor* GBL receptor (ScbR) activating the expression of *gfp* suggesting that such cross-phylum interactions are not impossible. However, in our case it is the contrary observation that does not find support in existing literature. It is an interesting and important observation for both the biosensor strains as well as Actinobacteria, and therefore needs further validation. In our opinion, we believe that such cross-taxa screening strategies might lead to discovery of newer molecules.

## Screening for Quorum Sensing in Actinobacteria: Limitations and Improvements

The lack of good biosensor system(s) which can respond to a very low quantity and a range of signaling molecules is a major limitation. Quorum sensing can be conclusively demonstrated only upon the isolation of the signaling molecule, followed by its structural determination and its ability to regulate phenotypes when added externally in the medium. However, Actinobacteria, such as *Streptomyces* cultures generally produce very low quantity of GBLs and its purification typically requires organic extraction of large (e.g., >400 L) volumes of spent culture medium. The existing sensor strains neither respond to such low quantity nor the range of GBLs produced, especially with longer C2 side chains. It is probably the main reason why the structure of only a few GBLs are known. In fact for these technical and economic reasons, Healy et al. (2009) did not determine the structure of GBL from *S. acidiscabies* and instead used an alternative strategy to indirectly prove the interaction of GBL with its cognate receptor (see below). In stark contrast, the AHL-responsive biosensor strains, such as *Chromobacterium violaceum* CV026 (McClean et al., 1997) and *gfp*-based recombinant *Escherichia coli* biosensor strains containing plasmids pJBA89, pJBA130, and pJBA132 (Andersen et al., 2001) respond to a wide range of AHL compounds even in nano Molar quantities. Their availability has significantly increased the detection of AHL-mediated quorum sensing in Gram-negative bacteria. Given this, there is an immediate requirement for efforts to create a similar biosensor system that can detect a wide variety of GBLs. In order to move forward, the priority should be to generate more data from the known GBLs and the mechanisms they regulate. This new information will significantly add toward developing such wide-range GBL-responsive biosensors.

Not many Actinobacteria exhibit AI-2-mediated quorum sensing which is typical of many other Gram-positive organisms. However, this could be attributed to its sensitivity to high glucose and acidic pH in the culture medium both of which have a strong inhibitory effect. While screening for AI-2 activity in bifidobacterial culture supernatants, Sun et al. (2014) could not detect any activity in MRSc, i.e., the standard culture medium for bifidobacteria. MRSc contains 20 g/L glucose and the end products of the bifidobacterial metabolism on hexoses are mainly acetic and lactic acid. By testing *V. harveyi* BB170, a known AI-2 producer at different pH values, the authors concluded that acidic pH negatively affected detection. AI-2 activity was reduced to approximately 40% at pH 4, i.e., the pH observed in bifidobacterial supernatants, and at 0.25 g/L of glucose (Sun et al., 2014). In contrast, during assays for the signaling molecule response regulator, ArpA, only those that are acidic (pH ∼5) bind the autoregulator when tested; basic proteins did not (Willey and Gaskell, 2011). Hence, information on the sensitivity of existing signaling molecules is warranted. Once this information is generated, it likely to improve the existing screening strategies.

A more feasible approach is to search for homologs of the autoregulator receptor gene (Willey and Gaskell, 2011). As shown by Santos et al. (2012), these genes share a high degree of similarity within a given taxon and designing of degenerate primers to PCR amplify and sequence them is a better strategy. Using a similar strategy, we were able to sequence majority of quorum sensing gene homologs from the genus *Aeromonas* and show that the system is ubiquitously present across all the species in this genus (Jangid et al., 2007). With the advancements in sequencing technology and reduced costs, conducting metagenomic studies using a similar approach would be very easy and is likely to generate an enormous depth of information that is still hidden and untapped. However, such novel strategies must be followed with caution and utmost planning. Further, the mere presence of the gene is by no means an evidence of a functional signaling system. Hence, cloning of such structural genes in an expression system is the only way to confirm its activity. However, it is imperative that for such a strategy the intact functional protein much be obtained and later-on use the purified proteins for further investigation.

Recently, some new receptor-based methodologies have been described. To circumvent the issue of requiring large amounts of cultures, Yang et al. (2005) reported an alternative detection system using ScbR, the receptor protein from *S. coelicolor* and electrospray tandem mass spectrometry (ESI-MS/MS). Using the success of affinity capture technology in proteomics studies, the authors developed recombinant receptors of butanolides, such as such as ArpA from *S. griseus*, FarA from *S. lavendulae*, BarA from *S. virginiae*, and SpbR from *S. pristinaespiralis* and used them as affinity capture molecules to trap butanolides, followed by MS analysis for identification. This method allows the isolation of butanolides from as low as 100 ml of *S. coelicolor* culture broth. In addition, it enables the detection of quorum sensing system in cases where the interaction between the signaling molecule and its cognate receptor is inhibited in acidic pH and high glucose. For instance, Healy et al. (2009) detected fragment ions bound by the purified GBL receptors from *S. acidiscabies*. These ions showed masses that were consistent with molecules possessing lactone functional groups such as those found in GBL compounds. This strategy might therefore be useful for strains with identified GBL receptors but where the interactions could not be proven.

The availability of a diverse set of biosensor plasmids is likely to increase the frequency of detection of Actinobacterial quorum sensing systems. Recently, Yang et al. (2009) developed a *gfp*-containing *E. coli-*based cell-free system for detecting GBL in *Streptomyces.* In this ScbR quorum sensing system, the *gfp* is fused downstream of the DNA binding site for the *S. coelicolor* GBL receptor, ScbR. The presence of purified His-tagged ScbR and cognate GBL results in fluorescence. This system allows to circumvent the issues of cell wall penetration and can be used for high-throughput screening as it allows assays to be completed within 4 h. Further, the protein–ligand interaction can easily be monitored without the use of radioisotopes and acrylamide gels. Similarly, Hsiao et al. (2009) used ScbR and its target DNA to control the expression of a kanamycin resistance gene in the presence of its cognate GBL. This new sensitive reporter system also allows detection of small quantities of GBLs and those that are difficult to detect. The authors propose that by altering the timing for extract preparation from cells, the detection of other GBLs could be enhanced from different strains. The kanamycin bioassay is likely to facilitate large-scale screening of GBL producers due to its higher sensitivity toward wide range of GBLs than the commonly used bioassay.

While these new approaches are likely to facilitate the discovery of additional GBLs, one important limitation is that most are targeted to detecting GBLs from Actinobacteria, especially *Streptomyces*. Hence, detailed investigation of other non-GBL mediated quorum sensing systems is required to gain insight into the mechanisms involved and thereby develop strategies for expanding the array of signaling molecule detection.

## Quorum Quenching Activity in Actinobacteria

With constant rise in the number of antibiotic-resistant bacteria, there is a need to look for alternative strategies to control their spread. Since most pathogens regulate their virulence by quorum sensing, it has become the most sought-after alternative target to control their spread. Chemical inactivation of the Gram-negative AHLs via alkaline hydrolysis is known for quite some time. However, the enzymatic degradation of signaling molecules is now the most researched field in quorum quenching to limit the growth of many animal and plant pathogens. Quorum quenching enzymes act in either of the two ways: (1) analogous to the chemical ring hydrolysis, acyl-homoserine is generated by AHL lactonases; and (2) the amide bond is degraded by AHL acylases. Screening for these enzymes in different ecosystems have shown great potential. For instance, AHL-degrading bacteria may make up 5–15% of the total cultivable bacteria in the soil and rhizosphere (Latour et al., 2013). Although small, it is a non-negligible and an important resource for developing biocontrol formulations. Screening for such enzymes has therefore become increasingly important.

The ability of Actinobacteria to produce the innumerable secondary metabolites, enzymes, and commercially important biomolecules has attracted researchers to explore this phylum for their role in quorum quenching activity. Endophytic actinomycetes and their AHL-lactonase enzymes have shown great potential in this regard (Chankhamhaengdecha et al., 2013). The authors made a first attempt toward screening for quorum quenching enzyme-producing actinomycetes from soil and plant tissues. With 51.5% of the tested strains possessing the quorum quenching activity, endophytic actinomycetes possessed the activity at higher frequency than the soil isolates at 36.9% demonstrating a great diversity and abundance of AHL-degrading actinomycetes. While one would think that quorum quenching is most useful for organisms that produce the signals enabling them to use them as a source of energy and nitrogen source (Flagan et al., 2003), organisms that do not produce the signals are also known to quench them, presumably to gain an advantage over communicating bacterial species in the same ecological niche (Wuster and Babu, 2008). For example, *Rhodococcus* and *Microbacterium* can degrade AHL signals without having any known ability to produce them. In fact, there is no evidence of quorum sensing for the latter, not even bioinformatic. Hence, more of such environmental screening studies that target Actinobacteria are warranted.

Specific members of the phylum *Actinobacteria* have also shown considerable potential in agro-environment due to their quorum quenching activity. Several Actinobacteria have the ability to colonize plant surfaces and thereby exclude plant pathogens either by competition or through inhibition by antibiotic production (Selvakumar et al., 2014). Over the last decade, a total of six actinobacterial genera: *Arthrobacter* (Flagan et al., 2003), *Microbacterium* (Wang et al., 2012), *Mycobacterium* (Chen and Xie, 2011), *Nocardioides* (Yoon et al., 2006), *Rhodococcus* (Park et al., 2006; Latour et al., 2013), and *Streptomyces* (Chen and Xie, 2011; Ooka et al., 2013) have been reported for their quorum quenching activity. Members of these genera known to exist as plant symbionts or as endophytes residing within plant hosts without causing disease symptoms are reported to produce AHL-inactivating enzymes. In fact, *Rhodococcus* has an unusually high number of AHL-inactivating lactonases (Wuster and Babu, 2008), that may play a role in the intracellular metabolism of lactone compounds such as GBL (Uroz et al., 2005). Due to its high AHL-degrading activity, *R. erythropolis* strain R138 has been used as a biocontrol agent to prevent soft-rot in plants. Genetic evidence suggests that the lactone catabolic pathway in the strain may not be the only pathway for AHL-inactivation. In addition, it possesses multiple homologs of various catabolic enzymes, thus enhancing the species’ metabolic versatility (Latour et al., 2013). Recently, two more strains of *R. erythropolis*, PR4 and MM30 of marine origin have been reported to enzymatically degrade *N*-oxododecanoyl-L-homoserine lactone (Romero et al., 2011). Similarly, the soil isolate *Nocardioides kongjuensis* A2–4^T^ is able to grow with N-hexanoyl-L-homoserine lactone as the sole carbon source suggesting that its quenching ability is worth exploration against plant pathogens (Yoon et al., 2006). Further, the AHL-degrading lactonase enzyme activity was also reported from the potato leaf-associated *Microbacterium testaceum* StLB037 (Wang et al., 2012). Recently, *Arthrobacter* species have been reported to inhibit quorum sensing in a cross phylum interaction (Igarashi et al., 2015). The strain PGVB1 produces arthroamide and turnagainolide that showed potent inhibition of *agr-*signaling pathway of quorum sensing in *Staphylococcus aureus* at 5–10 μM without showing cell toxicity. Similarly, a major metabolite piericidin A1 secreted by *Streptomyces* sp. TOHO-Y209 and TOHO-O348 demonstrated quorum sensing inhibiting activity against *C. violaceum* CV026 (Ooka et al., 2013). The piericidin class of metabolites are known inhibitors of NADH-ubiquinone oxidoreductase, with A1 specifically inhibiting both mitochondrial and bacterial NDAH- ubiquinone oxidoreductases. These studies suggest that Actinobacteria offer a unique system which, if exploited well, is likely to play a major role in controlling the spread of plant and human pathogens.

## Conclusion

The enormous metabolic and phylogenetic diversity that exists in Actinobacteria offers a unique opportunity to explore its multifactorial abilities for biotechnological applications. Quorum sensing is one such property that is evidently under-explored in this phylum. Based on the limited information that is known, quorum sensing systems in Actinobacteria show considerable diversity in terms of the types of signals and the mechanisms it controls. However, there exists a taxa specific segregation within the phylum. For instance, GBL-mediated regulation is not only limited to *Streptomyces* but is also species specific. Interspecific signaling is therefore likely to expand the list of compounds and mechanisms involved in quorum sensing. The lack of good detection systems is a major limitation for further exploration of the communication system in Actinobacteria. Developing newer systems which can respond to a wider range of signals and that too at very low quantities are the need of the hour. Further exploration using these systems within and between multiple taxa is likely to reveal an even greater diversity of signals. Similarly, the quorum quenching ability of Actinobacteria exhibit a great potential, especially through their use as bio-control agents for plant pathogens and in controlling the spread of antibiotic-resistant organisms. However, systematic screening of specific ecosystems is required to fully exploit the quorum quenching potential. Using the knowledge gained from an in-depth understanding of the existing quorum sensing systems, Actinobacteria are likely to exhibit a wider array of properties that are likely to have significant implications for plant, animal and human health.

## Author Contributions

KJ and AP designed the review. SM and UP did the referencing, preliminary sequence analysis, and compilation of data. KJ and AP finalized the structure of the review, analyzed the sequence data, and wrote the review.

## Conflict of Interest Statement

The authors declare that the research was conducted in the absence of any commercial or financial relationships that could be construed as a potential conflict of interest.
