# Supplementary material for: Quorum Sensing: An Under-Explored Phenomenon in the Phylum Actinobacteria
Source: Front Microbiol. 2016 Feb 10;7:131. doi: 10.3389/fmicb.2016.00131 (PMC4748050; doi:10.3389/fmicb.2016.00131)
Supplement: Supplementary file 1 [file Data_Sheet_1.DOCX]

Supplementary Material

Quorum sensing: an under-explored phenomenon in the phylum *Actinobacteria*

Ashish V Polkade, Shailesh S Mantri, Umera J Patwekar, Kamlesh Jangid*

*** Correspondence:** Corresponding Author: [jangidk@nccs.res.in](mailto:jangidk@nccs.res.in); [jangidk@gmail.com](mailto:jangidk@gmail.com)

# Supplementary Data

**Data File 1. Fasta file of sequences of 54 type species used in Fig. 1.** Sequence Accession numbers for type strains were taken from the LPSN website (<http://www.bacterio.net/>). Sequences were then downloaded in Fasta format from NCBI Nucleotide database and used for further analysis.
